# Acyclic Diterpene Phytol from Hemp Seed Oil (*Cannabis sativa* L.) Exerts Anti-Inflammatory Activity on Primary Human Monocytes-Macrophages

**DOI:** 10.3390/foods11152366

**Published:** 2022-08-07

**Authors:** Carmen M. Claro-Cala, Elena Grao-Cruces, Rocio Toscano, Maria C. Millan-Linares, Sergio Montserrat-de la Paz, Maria E. Martin

**Affiliations:** 1 Department of Pharmacology, Pediatric and Radiology, Faculty of Medicine, Universidad de Sevilla, Av. Sanchez Pizjuan s/n, 41009 Seville, Spain; 2 Department of Medical Biochemistry, Molecular Biology and Immunology, School of Medicine, Universidad de Sevilla, Av. Sanchez Pizjuan s/n, 41009 Seville, Spain; 3 Department of Cell Biology, Faculty of Biology, Universidad de Sevilla, Av. Reina Mercedes s/n, 41012 Seville, Spain

**Keywords:** phytol, nutraceuticals, unsaponifiable, hemp seed oil, functional foods, immunonutrition

## Abstract

Seeds from non-drug varieties of hemp (*Cannabis sativa* L.) have been used for traditional medicine, food, and fiber production. Our study shows that phytol obtained from hemp seed oil (HSO) exerts anti-inflammatory activity in human monocyte-macrophages. Fresh human monocytes and human macrophages derived from circulating monocytes were used to evaluate both plasticity and anti-inflammatory effects of phytol from HSO at 10–100 mM using FACS analysis, ELISA, and RT-qPCR methods. The quantitative study of the acyclic alcohol fraction isolated from HSO shows that phytol is the most abundant component (167.59 ± 1.81 mg/Kg of HSO). Phytol was able to skew monocyte-macrophage plasticity toward the anti-inflammatory non-classical CD14^+^CD16^++^ monocyte phenotype and toward macrophage M2 (CD200R^high^ and MRC-1^high^), as well as to reduce the production of IL-1β, IL-6, and TNF-α, diminishing the inflammatory competence of mature human macrophages after lipopolysaccharide (LPS) treatment. These findings point out for the first time the reprogramming and anti-inflammatory activity of phytol in human monocyte-macrophages. In addition, our study may help to understand the mechanisms by which phytol from HSO contributes to the constant and progressive plasticity of the human monocyte-macrophage linage.

## 1. Introduction

Hemp seeds (*Cannabis sativa* L.) of non-drug varieties have been widely used for many years as a source of medicines, foods, or fibers [1,2,3,4,5,6]. Compared to other vegetable oils, hemp seed oil (HSO) offers many benefits for the food industry, as an ingredient itself, or as an additive to increase the quality of animal-derived food products [2,3]. Additionally, regarding the ratio omega-6/omega-3 (3:1) of linoleic and linolenic acids, two essential polyunsaturated fatty acids (PUFAs) for human nutrition, HSO is considered to be remarkably balanced [7]. In line with our investigations of medicinal and food plants as a source of secondary metabolites with bioactivity, our group has recently evaluated a preliminary analytic screening of HSO [8]. The composition of HSO includes terpenes, phytosterols, and some tocopherols with remarkable anti-oxidative activity to scavenge free radicals that may have a role in certain signaling pathways related to the regulation of the inflammatory response [9,10,11]. Therefore, HSO-related compounds emerge with an anti-inflammatory and immune-modulatory role as a novel added value of HSO [2,3].

Conventional food waxes contain acyclic fatty alcohols, which have been isolated using saponification and solvent extraction from spinach, sugarcane, or beeswax [12]. These alcohols are responsible for health benefits, including reduction of cholesterol levels, platelet aggregation, or endothelial damage [13]. Phytol (3,7,11,15-tetramethylhexadec-2-en-1-ol) is one of those acyclic diterpene alcohols present in the composition of the essential oils of some aromatic plants and in the unsaponifiable fraction of vegetable oils [14]. The most abundant compounds in the unsaponifiable fraction of HSO are β-sitosterol, campesterol, phytol, cycloartenol, and γ-tocopherol [8]. Previous data report many therapeutic activities of phytol against mycobacteria and as an anticonvulsant, antispasmodic, or antitumoral agent [15,16,17]. Although some data point to the promising anti-inflammatory activity of diterpenes [14,18,19,20,21], little is known about the bioactivity of phytol [22]. In addition, although it is a major component of the unsaponifiable fraction of HSO, the biological activity in primary human monocytes of isolated HSO phytol has not been studied to date.

Both monocytes and macrophages appear to be notable therapeutic targets as the main sources of pro-inflammatory and anti-inflammatory cytokines, contributing to oxidative-mediated inflammatory cascades that occur during all stages of inflammatory disorders such as atherosclerosis [23,24,25]. In humans, monocyte cells are classified in classical monocytes (CD14^++^CD16^−^), intermediate monocytes (CD14^++^CD16^+^) and non-classical monocytes (CD14^+^CD16^++^) [26,27,28]. The highest proportion (around 85% of the total) of monocytes is classical monocytes. These cells behave like expert phagocytes, greatly express CCR2, and give rise to the M1 macrophage phenotype, which causes reactive oxygen species and cytokines (TNF-α, IL-1β, and IL-6) during inflammation or infection in response to LPS [29,30]. On the other hand, intermediate monocytes secrete pro-inflammatory cytokines and highly express CCR5, CD163, TLR4, and HLA-DR during activation [31]. Finally, non-classical monocytes are smaller and less granular cells, showing lower expression of CCR2 compared to classical or intermediate monocytes [31], but rich in CD16. Non-classical monocytes tend to the anti-inflammatory phenotype of polarizing M2 macrophages after various stimuli, such as IL-4, and play a role in patrolling, tissue repairing, and wound healing [30,32]. The purpose of this paper is to examine the effects of phytol, the acyclic diterpene alcohol isolated from the unsaponifiable fraction of HSO, on the activation of primary monocytes and mature macrophage human cells as indicators of inflammatory disorders.

## 2. Materials and Methods

### 2.1. Isolation of Phytol from an Unsaponifiable Fraction of Hemp Seed Oil

Following conventional protocols, from 1 Kg of HSO (Naturgreen, Murcia, Spain), the unsaponifiable fraction was isolated. The composition of this fraction was analyzed as described in Montserrat-de la Paz et al. using the IUPAC method [33]. Ethanol was used for the extraction of the acyclic alcohol fraction three times at room temperature, removing the solvent under vacuum conditions, to obtain a residue (225 mg) that was suspended using water, to continue the extraction with petroleum ether and chloroform. Using silica gel column chromatography, the residue from the chloroform layer was fractioned with eluents CHCl_3_–Me_2_CO, followed by purification using exclusion resin (RP C18 Si-gel column) with eluent CH_3_OH–water (8:2–7:3). The GC-MS study was assessed using a capillary gas chromatograph HP 5890 Series II Plus connected to a mass spectrometer HP 5972 (both models from Hewlett-Packard). Operating conditions and equipment were as follows: a capillary column (30 m by 0.25 mm internal diameter, Hewlett-Packard) coated with HP silicon 5% phenyl methyl, an oven temperature increasing 30 °C min^−1^ from 60 °C to 130 °C, followed by 4 °C min^−1^ rising from 130 °C to 300 °C, a carrier gas pressure (He) fixed until the end of the temperature program at 1.05 × 105 Pa, increasing then at 0.04 × 10^5^ Pa min^−1^ from 1.05 × 10^5^ to 1.5 × 10^5^ Pa, temperature of 50 °C for injector (on column), 70 eV of electron energy, 170 °C of source temperature and 1.5 s of cycle time. To confirm the purity grade, the isolated phytol from HSO was analyzed by GC compared to the phytol standard (>97% purity, Sigma-Aldrich, Madrid, Spain). 

### 2.2. Blood Collection Procedure and Human Monocytes Isolation

The “Centro Regional de Transfusiones Sanguíneas y Banco de Tejidos de la province de Sevilla y Huelva” provided samples from which peripheral blood mononuclear cells (PBMC) were obtained from buffy coats after centrifugation using a Ficoll-Histopaque gradient (Sigma). According to the manufacturer’s instructions, with a midiMACS system (Miltenyi Biotec, Madrid, Spain), monocyte cells were isolated from PBMC using CD14 microbeads and LS columns. By flow cytometry (FACScanto II flow cytometer and FACSDiva software, BD), the purity for CD14 monocyte isolations was quantified routinely at >90%. After isolation, monocyte cells became resuspended in RPMI 1640 medium, in which 10% heat-inactivated fetal bovine serum, penicillin, streptomycin, and L-glutamine were added [34].

### 2.3. FACS Immunostaining of Circulating Monocytes Cells

Using flow cytometry, membrane expression of CD14 (APC-Cy7 anti-human CD14, Miltenyi), CD16 (PE anti-human CD16, Miltenyi), and CCR2 (APC anti-human CCR2, Vitro) in circulating monocytes were analyzed. After in vitro stimulation in the presence or absence of 100 ng/mL LPS, 5 × 10^5^ purified monocytes cells were treated for 24 h with 10–100 mM phytol, according to the manufacturer’s instructions. The cells were incubated at room temperature, in the dark, for 15 min, with antibodies, and finally, erythrocytes were removed using FACS lysing solution (BD). Using a FACSCanto II flow cytometer (BD) calibrated with FACSDiva software (BD), the mean fluorescence intensity (MFI) was quantified. For each sample, the MFI of 10^4^ counted cells was tested. Monocyte cells became gated as forward scatter^high^ (FSC^high^)-side scatter^high^ (SSC^high^) cells. Expression levels appear as MFI values corrected with isotope control antibodies for nonspecific binding.

### 2.4. Differentiation and Polarization of Monocyte-Derived Macrophages M1 and M2

To differentiate into M0 macrophages, for 6 days in the presence of 25 ng/mL of human recombinant M-CSF, monocytes were induced. Every 2 days, the culture medium was replaced with fresh medium. Using flow cytometry (>95%) and CD68 antigen (anti-human CD68 monoclonal antibody, Miltenyi Biotec, Madrid, Spain), we determined the degree of differentiation of the resulting cells. To obtain M1 or M2 polarized macrophages, additional treatment of M0 macrophages was evaluated for 24 h with LPS (100 ng/mL) and IFNγ (20 ng/mL) or with IL-4 (20 ng/mL) [35]. Finally, M0 macrophages were exposed to 10–100 mM phytol for 24 h to determine the effects of the polarization process. As a positive control, 1 µM of dexamethasone (Sigma, Madrid, Spain) was used (data not shown).

### 2.5. MTT Cell Viability Assay 

First, 1 × 10^5^ monocytes cells/well were seeded in 96-well plates and then differentiated into M0 macrophages. Afterward, macrophages were incubated for 24 h with or without 10–100 mM phytol. Finally, incubation of cells with MTT solution (Sigma) was assessed until a purple precipitate formed. MTT-formazan crystals were solubilized using DMSO (Sigma) before quantification with a microplate reader at 570 nm corrected to 650 nm [36]. When these values were compared with those of nontreated cells as controls, the survival of the cells was displayed as the percentage of absorbance. 

### 2.6. Isolation of RNA and qRT-PCR Protocol 

According to the manufacturer’s instructions, Trisure Reagent (Bioline) was used for the extraction of total RNA. To assess the quality of RNA, the ratio A_260_/A_280_ obtained in a NanoDrop ND-1000 Spectrophotometer (Thermo Scientific, Madrid, Spain) was used. The first step in this process was to subject 1 µg RNA to reverse transcription (iScript, Bio-Rad, Madrid, Spain); 20 ng of the obtained cDNA was used as a template for real-time PCR amplifications. Using a CFX96 system (Bio-Rad), mRNA levels were analyzed for specific genes. In every PCR reaction, the cDNA template was mixed with a Brilliant SYBR green QPCR Supermix (Bio-Rad) in which glyceraldehyde 3-phosphate dehydrogenase (GAPDH) as the housekeeping gene or the primer pairs for either gene were present (Table 1). PCR amplifications were performed in triplicate, and to quantify the relative levels of mRNA expression for every gene tested, the average threshold cycle (Ct) numbers of triplicates were used. With the standard 2^−(ΔΔCt)^ method, the proportion of change in mRNA expression in candidate genes was quantified. Data were expressed as percentages adjusted to controls and normalized to endogenous reference gene content [37]. 

### 2.7. Cytokine Release Measurement

Following the manufacturer’s indications, we quantified the levels of TNF-α, IL-6, and IL-1β cytokines in culture supernatants using the ELISA (enzyme-linked immunosorbent) assay (Diaclone, Besancon, France). In each assay, cytokine concentrations were expressed in pg/mL after serial dilution of human recombinant standards were calculated in calibration curves. 

### 2.8. Data Analysis 

Data are expressed as arithmetic means ± standard deviations (SD). Values were estimated using version 6.01 of the Graph Pad Prism software (San Diego, CA, USA). In each parameter, the statistical importance of variances among the groups was analyzed using a one-way ANOVA analysis of variance, following the Tukey multiple comparison post-hoc test. Any *p*-value < 0.01 was determined statistically relevant.

## 3. Results and Discussion

### 3.1. Chemical Characterization of HSO

**Figure 1:**
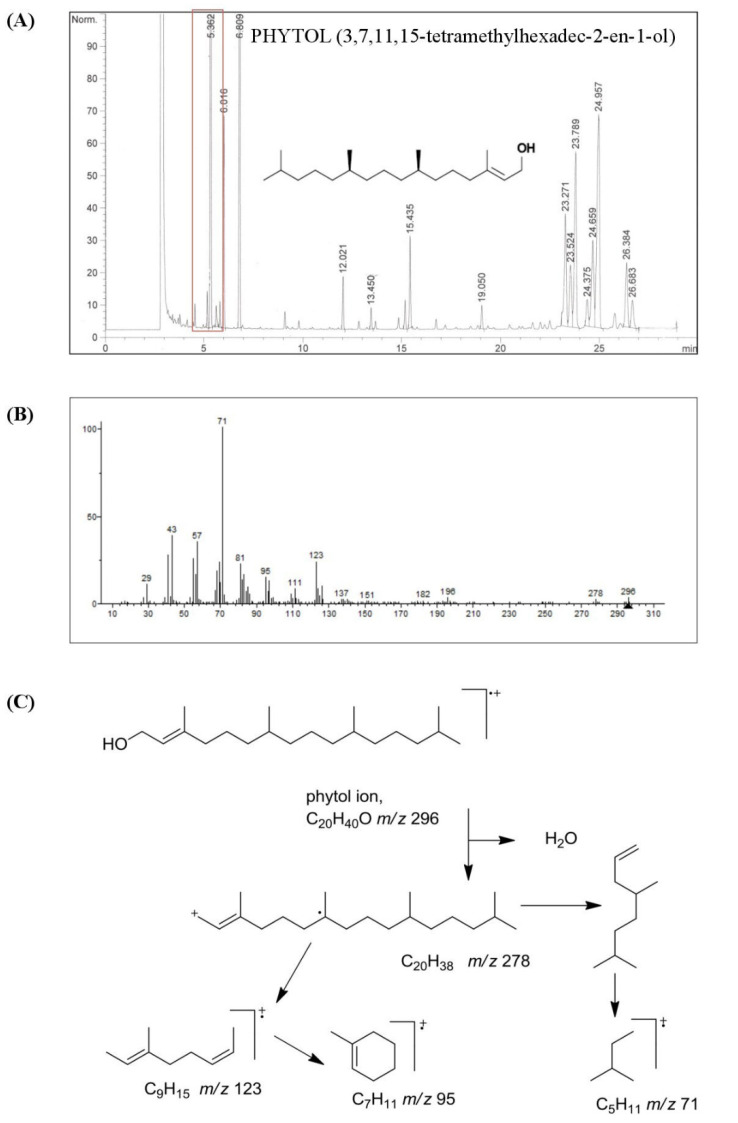
Characterization of phytol isolated from HSO. (**A**) Gas chromatogram of acyclic aliphatic alcohols, triterpene alcohols, and 4-methylsterol fractions of unsaponifiable HSO and chemical structure of phytol (3,7,11,15-tetramethylhexadec-2-en-1-ol). (**B**) Mass spectrum and (**C**) schematic representation of the phytol mass fragmentation pattern.

Our study performed the isolation and characterization of the acyclic fatty alcohol fraction from an unsaponifiable matter of HSO. On the other hand, and for the first time, we explored the effect of phytol, the major compound in this fraction, on both the inflammatory response and reprogramming of functional phenotypes in human primary monocyte-derived mature macrophages. As shown in Figure 1, the main acyclic alcohols present in HSO were phytol (73.57%), followed by geranylgeraniol (11.45%) and hexacosanol (7.05%), representing 92% of the total fraction (226.94 ± 2.26 mg/kg of HSO). These components are quite different from those of policosanol, which is a commercial mixture of very long-chain alcohols containing mostly octocosanol (~60%), triacosanol (~13%), and hexacosanol (~6%) [38], and other vegetable oils such as olive oil, pomace olive oil, grape seed oil, and evening primrose oil [12,39,40]. 

To date, several chemical and nutritional studies have reported that acyclic fatty alcohols obtained from waxy materials of different origins exert beneficial effects on physiology [13,41]. For example, fixed oil has been described as having beneficial effects on osteoarthritis [42], hyperlipidemia [43], hyperalgesia [44], and inflammation [45,46]. However, little research has dealt with the pharmacological effects of phytol (3,7,11,15-tetramethylhexadec-2-en-1-ol), the main compound (167.59 ± 1.81 mg/kg of HSO) present in the acyclic fatty alcohol fraction isolated from HSO [16]. Phytol is scarcely present in human foods, such as asparagus, beans, spinach, or raw vegetables [47].

Free phytol in those products was reported in the range of 0.7–2 mg/kg of undried food. In dried tea or beef stock cubes, higher amounts of (12.5–14.7 mg/kg) phytol have been reported [48]. To date, previous studies have demonstrated the low toxicity and high tolerance of phytol, increasing the interest in this natural bioactive compound from plants [14,16,49]. Many data support the bioactivity of phytol in vivo and in vitro [50,51,52]. Our study demonstrates that HSO is one of the phytol-richest natural products.

### 3.2. Effect of Phytol on Monocyte Differentiation and Macrophage Polarization

**Figure 2:**
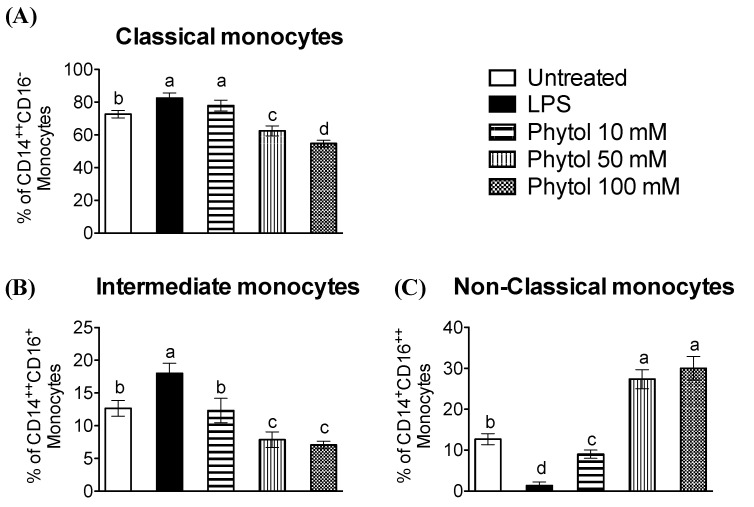
Effect of phytol on monocytes cells. FACS analysis (MFI) data of CD14 and CD16 as monocyte surface markers after 24 h treatment in the presence or absence of 100 ng/mL of LPS and phytol at 10, 50, or 100 mM. (**A**) Classical monocytes CD14^++^CD16^−^, (**B**) intermediate monocytes CD14^++^CD16^+^, and (**C**) non-classical monocytes CD14^+^CD16^++^. Values are shown as means ± SD (n = 3) and, when marked with different letters, are significantly different (*p* < 0.01).

The literature on HSO has already highlighted the value of this natural product for the identification and medicinal use of its components, including phytol. For that reason, it is necessary to support the promising advantages of HSO in the immunology area or in inflammatory and vascular-related dysfunctions where one of the pivotal cells for the initiation of inflammation is the monocyte [53,54,55]. Most circulating monocytes are known as classical monocytes (CD14^++^CD16^−^). Other subsets are non-classical monocytes (CD14^+^CD16^++^) and intermediate monocytes [26,27]. Both classical and intermediate monocytes have a pro-inflammatory phenotype that, as a response to LPS treatment, quite actively produces TNF-α, IL-1β, and IL-6, thus promoting various inflammatory disorders, including atherosclerosis [56,57]. Consequently, to avoid continuous inflammatory events and complete controlled repair, the correct balance of the monocyte cell subsets appears to be critical. There is a significant increase in CD14 expression in classical and intermediate monocytes when comparing LPS-treated cells with control untreated cells (Figure 2A,B). The addition of phytol overrode the LPS effect diminishing CD14 expression and also remarkably increasing CD16 (Figure 2C). The results of our study point, for the first time, to the feasible role of phytol as a regulator in human monocytes of the balance among the different subsets of monocytes. Classical and intermediate cells with pro-inflammatory phenotype show lower surface expression, while non-classical monocytes exhibit higher CD16 values after phytol doses compared to LPS. Therefore, phytol appears to be a promising indicator for preventing persistent inflammation and achieving controlled repair.

**Figure 3:**
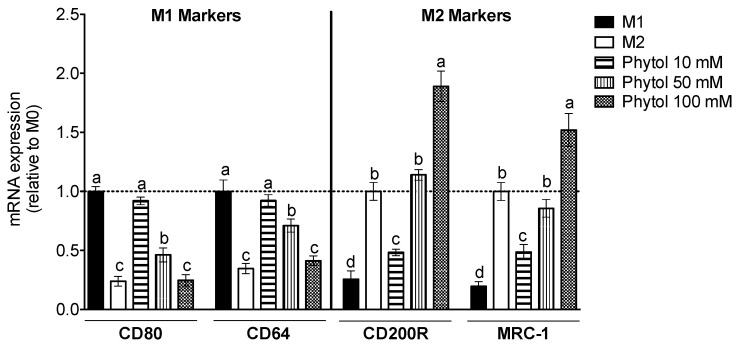
Effect of Phytol on polarization into M1 or M2 macrophages. M0 macrophage cells were treated with LPS and IFNγ for the M1 control, with IL-4 for M2 control, or with 10–100 mM phytol for an additional 24 h. RT-qPCR was used to measure the relative gene expression of the markers CD80, CD64, CD200R, and MRC-1. The values are means ± SD (n = 3) and, when marked with different letters, they are significantly different (*p* < 0.01).

In response to specific local microenvironments, tissue-infiltrated monocyte-derived macrophages undergo polarization into M1, pro-inflammatory type 1, or M2, anti-inflammatory type 2 cells, both of which play a critical role in early events and subsequent resolution of the immune response [23,57]. These M1 and M2 macrophages illustrate the extremes of a continuum of functional phenotypes [27,58]. On the one hand, M1 macrophages originate from LPS and/or IFNγ, while the phenotype of M2 macrophages is influenced by IL-4. Therefore, macrophage plasticity modulation appears to be a potential strategy to control joint inflammation. The phytol has previously been observed to decrease acute inflammation in vivo and pro-inflammatory cytokine production in peritoneal exudate and neutrophils from mice [14,59,60]. Therefore, we asked whether phytol had any effect on polarization and cytokine response in human mature macrophages. For that purpose, we quantified the gene expression of phenotypic macrophage markers for M1 (CD80, CD64) and M2 (CD200R, MRC-1). As shown in Figure 3, both CD80 and CD64 genes appear to be up-regulated by LPS plus IFNγ, while CD200R and MRC-1 appear to be up-regulated by IL-4. It was noteworthy to observe that phytol skewed macrophage polarization towards M2. 

### 3.3. Effects of Phytol on Pro-Inflammatory Cytokine Levels

**Figure 4:**
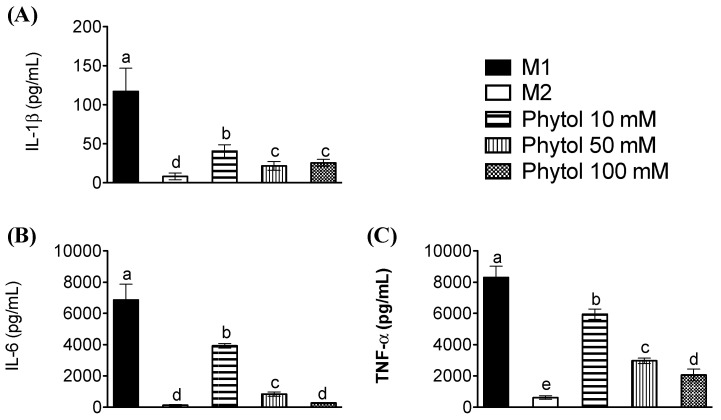
Effect of phytol on the release of pro-inflammatory cytokines. Cells from M0 macrophages were incubated in the presence of LPS and IFNγ, for M1 control, or with IL-4 for the M2 control, or with 10–100 mM Phytol for an additional 24 h. Using ELISA protocols, in polarized macrophage culture supernatants, the concentrations of (**A**) IL-1β, (**B**) IL-6, and (**C**) TNFα were measured. The values are means ± SD (n = 3) and, if marked with different letters, they are significantly different (*p* < 0.01).

This ability of phytol to regulate the polarization of M0 macrophage cells was additionally studied by analyzing the release into the culture medium of pro-inflammatory cytokines of the M1 phenotype, such as TNFα, IL-6, or IL-1β, upon exposure to LPS. Analysis of cytokine production (Figure 4A–C) illustrated that phytol significantly decreased the inflammatory capability of mature human mature macrophages. The quantification of pro-inflammatory cytokines in culture medium showed lower values than the control M1 when IL-1β, IL-6, and TNF-α were tested. Τhis tendency correlates with phytol doses, diminishing half the average values when comparing 10-, 50-, and 100 mM treatments. IL-6 release after 50 mM phytol is the lowest decrease, well over half of the 10 mM dose. Finally, the production of pro-inflammatory cytokines compared to M1 was decreased in the lowest proportion with 10 mM phytol and dramatically diminished with 100 mM phytol dose, as shown in Figure 4C.

Previous research findings have shown that macrophage-derived cytokines are crucial in human aging [61,62] and also in a wide range of human inflammatory disorders [63,64,65,66,67,68,69,70]. This phenotype is considered to operate in dynamic interchange if there is an extreme risk of inflammation risk and a negative feedback procedure is needed, for example, to stop inflammation [71,72]. These findings support the idea that phytol ingestion may prevent macrophage activation and alter macrophage plasticity in human tissue under inflammatory conditions.

## 4. Conclusions

These results showed that phytol, which was isolated and identified for the first time in HSO, can help to better understand the specific mechanism by which this acyclic diterpene exerts beneficial effects on monocyte-macrophage plasticity. So far, HSO has already been demonstrated to include healthy polyunsaturated fatty acids, as well as antioxidant tocopherols and anti-inflammatory phytosterols in its unsaponifiable fraction. In recent years, inflammation has emerged as a leading pathophysiologic mechanism in atherosclerosis and other diseases, so the effects of phytol on different hallmarks of the inflammatory response contribute to the recommendation of HSO as an interesting source of functional compounds.

## Figures and Tables

**Table 1:** Sequences of RT-PCR primers for gene expression analysis.

Target	GenBank Accession Number	Direction	Sequence (5′→3′)
CD80	NM_005191.3	Forward Reverse	GGGAAAGTGTACGCCCTGTA GCTACTTCTGTGCCCACCAT
CD200R	NM_138940.2	Forward Reverse	GTTGCCCTCCTATCGCATTA TGGAAATTCCCATCAGGTGT
MR	NM_002438.3	Forward Reverse	GGCGGTGACCTCACAAGTAT ACGAAGCCATTTGGTAAACG
CD64	NM_000566.3	Forward Reverse	GTCCAAATCTCCAAGTGCGG CCCAAGTATGAGAGCAGCGT
GAPDH	NM_001289746	Forward Reverse	CACATGGCCTCCAAGGAGTAAG CCAGCAGTGAGGGTCTCTCT

## Data Availability

Data is contained within the article.
